# Ensemble Modeling Reveals Severe Contraction of Dhole's ( *Cuon alpinus* Pallas, 1811) Suitable Habitat and Future Climate Refugia Across China

**DOI:** 10.1002/ece3.73822

**Published:** 2026-06-19

**Authors:** Taifu Huang, Shuaishuai Gao, Wei Cong, Jianhua Yu, Zhen Li, Yuan Yang, Tong Zhang, Yuan Wang, Zhonghua Wang, Jia Li, Yadong Xue, Yuguang Zhang

**Affiliations:** ^1^ Key Laboratory of National Forestry and Grassland Administration on Forest Ecosystem Conservation and Restoration Ecology and Nature Conservation Institute, Chinese Academy of Forestry Beijing China; ^2^ Central South Investigation and Planning Institute of State Forestry and Grassland Administration Changsha China; ^3^ Forestry Inventory and Planning Institute of Tibet Autonomous Region Lasa China; ^4^ Management Center of Yanchiwan National Nature Reserve Subei China; ^5^ Institute of Ecological Conservation and Restoration Chinese Academy of Forestry Beijing China

**Keywords:** climate change, conervation gap, dhole ( *Cuon alpinus* ), species distribution modeling (SDM), suitable habitat

## Abstract

Climate change not only poses a global biodiversity conservation challenge but also significantly reshapes wildlife habitat distributions. The dhole ( *Cuon alpinus* Pallas, 1811), a top predator within the mammalian order Carnivora, is classified as Endangered (EN) on the IUCN Red List and designated as a National Class I Protected Wildlife Species in China. This study analyzed past, current, and future suitable habitat dynamics for dholes in China using ensemble species distribution modeling, integrating occurrence records, and climate change projections. Our analysis revealed that the top three important variables were the min temperature of coldest month, elevation, and slope in the past period; however, isothermality, elevation, and slope in the current period. The current suitable habitat area for dholes in China is estimated to be 1,434,750 km^2^, primarily distributed around the Qinghai‐Tibet Plateau, China. Suitable habitat for dholes spanned mainly western, southern, and central China in the past. Overall, suitable habitat area declined by 47.59% between the past and current periods in China. This significant range contraction appears to be driven by a complex interplay of climatic and topographic factors. Under the climate change scenarios, a decline of 12.04%–13.12% in dhole suitable habitat is projected in China. The identified core area of future refugia were placed in the Kunlun‐Altun Mountains, Danghenan Mountains, Qilian Mountains, southeastern Tibet, and Hengduan Mountains. Despite conservation advancements, significant habitat deficits persist, necessitating urgent expansion of the protected area network and evidence‐based adaptive management in core zones. These results highlight the pressures of climate change on dhole persistence in China, emphasizing the need for spatially explicit, climate‐adaptive conservation strategies.

## Graphical abstract

The dhole ( *Cuon alpinus* ), an endangered carnivore species and one of China's highest‐level protected animals, faces significant threats from both climate change and human disturbance. Since the 1980s, dhole habitat has dramatically shrunk by approximately half, with the current main stronghold being the Qinghai‐Tibet Plateau. Key factors influencing their habitat include climatic, topographic, and anthropogenic factors. Future projections indicate a further significant habitat loss of 12.04%–13.12% by mid‐century (2050s). Critical future refuges where dholes might survive include mountain ranges like the Kunlun‐Altun, Qilian, Danghenan, southeastern Tibet, and Hengduan Mountains.

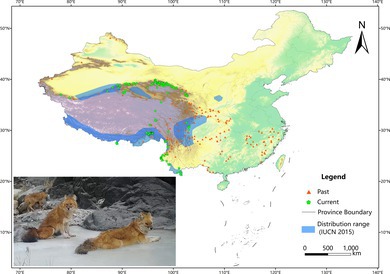

## 1 Introduction

Climate change and human activities significantly affected the global stability and balance of biodiversity (Nogués‐Bravo et al. 2018; Sunday 2020; Thompson et al. 2023), and profoundly influences the distribution and survival of wildlife (Risely et al. 2023; Cohen and Jetz 2025). Research shows that biological communities are responding to climate warming by migrating toward higher latitudes and elevations (Liu et al. 2025; Hoffmann et al. 2019). This shift has not only led to significant disruptions in trophic cascades within ecosystems, but may also exacerbate human‐wildlife conflicts (Lenoir et al. 2008; Bestion et al. 2019; Calhoun et al. 2025). Consequently, assessing the ecological niche responses of species to climate change has become a key research focus in conservation biology.

The dhole ( *Cuon alpinus* Pallas, 1811), a large canid within the order Carnivora, is classified as Endangered (EN) on the IUCN Red List and designated as a National Class I Protected Wildlife Species in China (Kamler et al. 2015; Wu et al. 2021). This social carnivore typically forms packs of 3–10 individuals, with larger aggregations of up to 25 individuals recorded (Kamler et al. 2015; Ma et al. 2023; Charaspet et al. 2020; Ghaskadbi et al. 2022). Historically distributed across Central, East, and South Asia, dholes occupied diverse habitats ranging from deserts to forests. The range extended northward to the Amur region and Lena River headwaters north of Lake Baikal (Russia), southward to Sumatra and Java (Indonesia), westward to Kazakhstan, and eastward to Anhui and Zhejiang provinces in eastern China (Smith and Xie 2009; Kamler et al. 2015; Liu et al. 2019). However, driven by habitat loss, retaliatory killing, disease, and prey depletion, population declines and range contraction mirror trends observed in most canid species globally (Thinley et al. 2021; Porto et al. 2024), with only a few exceptions, such as the golden jackal ( *Canis aureus* ), which has experienced population increases (Popovici et al. 2025).

Dholes have vanished from over 60% of their historical range (Wolf and Ripple 2017; Xia et al. 2023), with probable regional extinctions in Russia and Afghanistan (Kamler et al. 2015; Ghaskadbi et al. 2022). Encouragingly, although the population of dholes may be critically endangered, population recoveries have been documented in Nepal and India (Kazi et al. 2021; Vaghela 2024; Ghimirey et al. 2024). The currently recorded distribution areas include the Indian subcontinent, Southeast Asia, and China (Kamler et al. 2015; Tananantayot et al. 2022; Yang et al. 2025). In China, their distribution has dramatically contracted from historical records across most provinces to recent verified occurrences in Xinjiang, Qinghai, Gansu, Tibet, Sichuan, and Yunnan, based on camera trapping and molecular scatology (Liu et al. 2019; Liu, Wang, et al. 2024; Liu, Ji, et al. 2024; Shi et al. 2023). However, dhole research remains limited, primarily focusing on abundance, spatiotemporal ecology, diet, habitat, disease, and phylogeny (Punjabi et al. 2022; Srivathsa et al. 2023; Marciszak et al. 2023; Choki et al. 2023; Tananantayot et al. 2022). Most studies have originated from the Indian subcontinent and Southeast Asia, with scarce data from Central and East Asia.

Recently, Yang et al. (2025) modeled the current distribution of dholes in China using open‐access data. Meanwhile, current habitat suitability analyses have been conducted for the Qilian Mountains and Sichuan, China (Liu, Wang, et al. 2024; Liu, Ji, et al. 2024; Xia et al. 2023). However, these studies lack quantitative assessments of the impacts of future climate change on dhole habitat. Species Distribution Models (SDMs) have become critical tools for assessing habitat suitability, informing conservation planning, and predicting climate change impacts (Elith and Leathwick 2009; Hellegers et al. 2020; Bennington et al. 2024). For rare species with limited occurrence data, ensemble models like Biomod2 can often provide more robust predictions compared to individual algorithms (e.g., GLM, RF, MaxEnt) by integrating multiple modeling approaches and thereby reducing the uncertainty inherent in any single model (Thuiller et al. 2009; Hao et al. 2019, 2020). It is important to note, however, that the performance of ensemble models is contingent upon specific model settings, parameter configurations, and effective bias correction, and they do not universally outperform well‐tuned individual models in all circumstances (Marmion et al. 2009; Hao et al. 2019, 2020). For instance, in a study utilizing giant panda distribution data, Luo et al. (2017) demonstrated that the Biomod2 ensemble framework exhibited superior performance in both the area under the receiver operating characteristic curve (AUC) and accuracy compared to MaxEnt when the input occurrence records were relatively limited and under their specific modeling protocol. This case highlights the potential advantage of ensemble approaches under specific conditions of data scarcity.

Given the scarcity of dhole distribution data in China and the conservation implications of climate change, this study employs Biomod2 to evaluate past and current habitat suitability, quantify habitat changes, and project future climate impacts, providing critical insights for dhole conservation in China and globally. We aim to answer: (1) What is the current distribution status of suitable habitats for dholes in China? (2) What are the changing trends of suitable habitats under climate changes and the key influence factors? (3) Where are the core refuges for dholes during the future period of 2041–2060?

## 2 Materials and Methods

### 2.1 Data Collection

#### 2.1.1 Distribution Sites of Dholes

**FIGURE 1:**
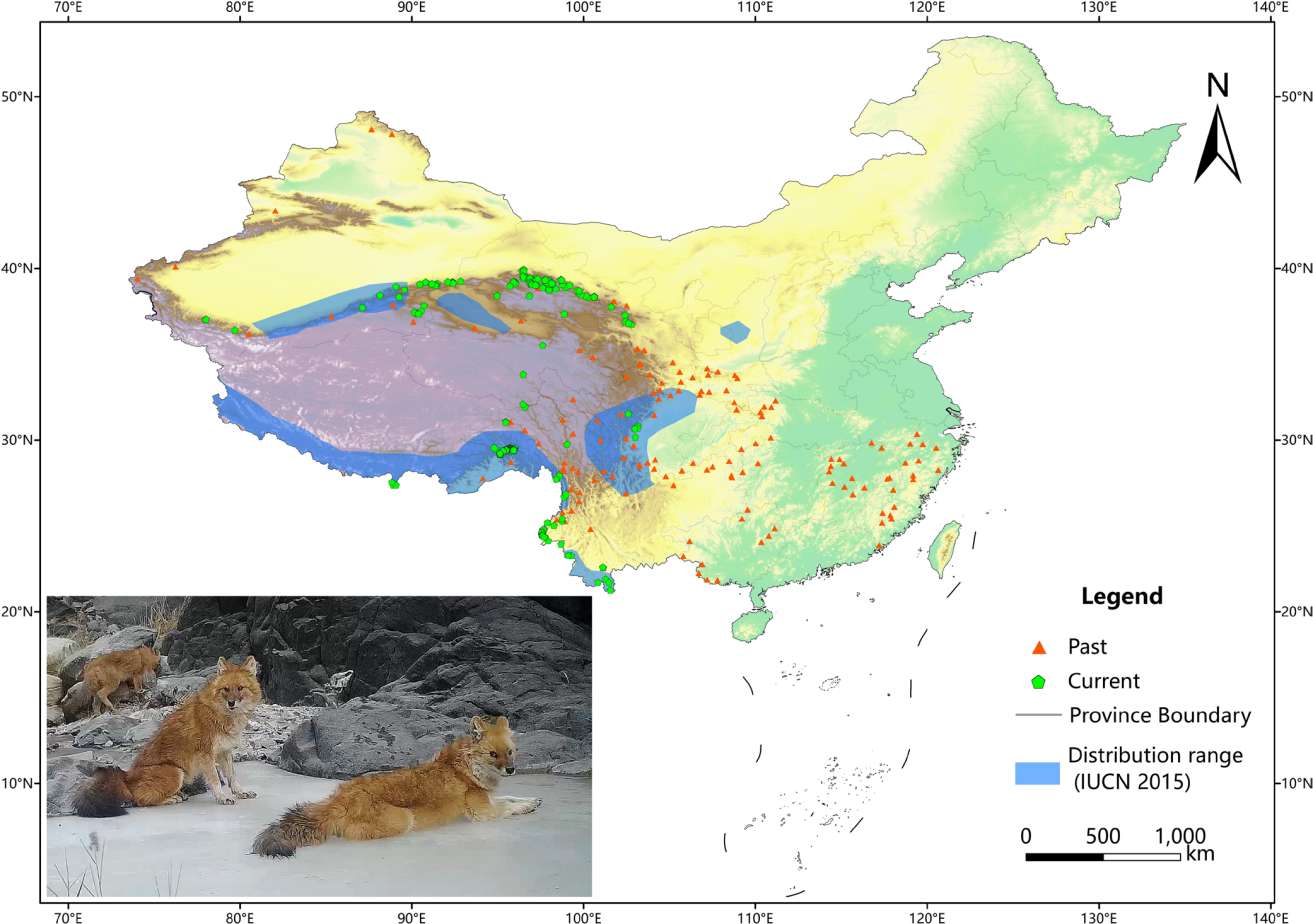
Dhole occurrence sites in past and current periods.

According to Ding et al. (2024), the distribution sites of dholes were categorized into the past and the current (Figure 1; Table S1). The past sites refer to records from 1980 to 2000, mainly obtained from the “Survey on key terrestrial wildlife resources in China” (Ma and Zhang 2009), which focused on the period 1995–2000 and provided detailed information on dhole distribution.

The current sites cover the period from 2001 to 2024, with data sourced from our field investigations, related scientific research, and news reports, resulting in a total of 364 current sites. Additionally, we downloaded the dhole's global species range map from the IUCN Red List of Threatened Species website (https://www.iucnredlist.org/) for comparative reference. To mitigate spatial autocorrelation from adjacent, overlapping, or duplicate records, we used ArcGIS 10.8 to spatially rarefy the distribution sites, retaining only one record within a 5.86 km radius—the average daily movement distance of dholes (Tananantayot et al. 2022). Finally, 145 past sites and 127 current sites were retained for analysis in the model. Additional details about occurrence data collection are available in the Supporting Information (Table S1).

#### 2.1.2 Environmental Variables

Based on existing research (Choki et al. 2023; Thing et al. 2022; Tananantayot et al. 2022), environmental factors influencing the habitat suitability of dholes are categorized into four groups: climatic factors, biological factors, human activity factors, and topographic factors.

1. Climatic Factors: Climate data includes the past, current, and future climate scenarios. The 19 bioclimatic variables (Bio1‐19) for past (1980–2000) and current (2001–2020) scenarios were derived from monthly aggregated minimum temperature, maximum temperature, and precipitation datasets spanning 1980–2020 from Worldclim (https://worldclim.org/). These variables were calculated using the biovars function in the R package ‘dismo’, following the methodology applied in Gao et al. (2025). Future meteorological data (2041–2060) were selected three general circulation models (BCC‐CSM2‐MR model, MIROC6, and IPSL‐CM6A‐LR), based on SSP1‐2.6, SSP3‐7.0 and SSP5‐8.5 emission scenarios from WorldClim, reflecting China's climate dynamics more accurately (Guo et al. 2024; Gao et al. 2025).
2. Biological Factors: These include Net Primary Productivity (NPP) and Normalized Difference Vegetation Index (NDVI). NPP data were sourced from MOD17A3HGF data at 500 m resolution, while NDVI data were derived from two datasets: AVHRR NDVI at 4 km resolution and MOD13A2 V061 NDVI at 500 m resolution. Mean values for past (1980–2000) and current (2001–2020) scenarios were calculated separately using Google Earth Engine, with each scenario based on aggregated climate data from its respective time period.
3. Human Activity Factors: These include land use type (GLC), human footprint index (Hfp), nighttime light index (Ntl), distance to roads (Road), and distance to railways (Railway). Land use type data is sourced from the GLC_FCS30D dataset (Zhang et al. 2021), and two periods (2000 and 2020) are used to represent the past and the current, respectively. Human footprint index (1 km resolution; Mu et al. 2022) and nighttime light index (0.5 km resolution; Chen et al. 2021) were acquired for past (2000) and current (2020) scenarios respectively. Distances to roads and railways for both past and current periods were extracted from the 2000 China Main Roads Dataset (http://geodata.pku.edu.cn) and the 2024 OpenStreetMap dataset (https://www.openstreetmap.org), respectively, with Euclidean distances calculated using ArcGIS 10.8 software.
4. Topographic Factors: These include elevation, slope, aspect, and distance to water sources. Elevation, slope, and aspect data are sourced from digital elevation models (DEM) with a resolution of 30 m, provided by the Geospatial Data Cloud (https://www.gscloud.cn/). Distance to water sources was extracted from the 2024 OpenStreetMap dataset's water systems, with Euclidean distances calculated using ArcGIS 10.8 software.

All environmental variables were reprojected to WGS_1984, resampled to a unified 5 km × 5 km resolution using bilinear interpolation, and converted to ASCII format. To reduce model overfitting caused by multicollinearity among environmental variables, we excluded one variable from any pair with a Pearson correlation coefficient |*r*| ≥ 0.80 by ENMTools R package (Figure S1; Gao et al. 2025). The retained variable was chosen based on its stronger ecological relevance to the study species (Liu, Wang, et al. 2024; Liu, Ji, et al. 2024; Yang et al. 2025). Ultimately, 9 low‐similarity and highly representative environmental variables were used (Table 1). Under future scenarios, climate variables were updated to raster layers corresponding to each period, while all non‐climatic variables (e.g., DEM, NDVI, and Slope) were held constant for future suitability predictions.

**TABLE 1:** Environmental variables used in this study.

Factor	Variables	Contribution (Past)	Contribution (Current)
Climatic Factor	Isothermality (Bio3)	6.64%	14.96%
	Min Temperature of Coldest Month (Bio6)	58.95%	4.59%
	Precipitation Seasonality (Bio15)	1.78%	3.24%
Biological Factor	Normalized Difference Vegetation Index (NDVI)	4.36%	2.53%
Terrain Factors	Elevation data are sourced from digital elevation models (DEM)	41.02%	54.71%
	Slope data are sourced from digital elevation models (Slope)	6.65%	10.48%
	Distance to water sources (Water)	1.03%	0.55%
Human Activity Factor	Human Footprint dataset (Hfp)	4.72%	0.83%
	Distance from railway (Railway)	1.73%	2.25%

### 2.2 Model Construction and Evaluation

This study constructed an ensemble species distribution model for Chinese dholes using the Biomod2 version 4.2–5. The Biomod2 platform includes 11 models, such as the Maximum Entropy Model (MaxEnt), Random Forest (RF), and Generalized Additive Model (GAM). For parameter settings, see Table S2. Nine selected environmental variables (Table 1) were input into the Biomod2 platform to simulate changes in suitable habitats for dholes under past, current, and future scenarios. Variable contribution rates were calculated within Biomod2 to quantify the relative influence of each environmental predictor, thereby identifying the key ecological drivers of dhole habitat suitability.

Due to the limitations in field surveys, only species presence data (occurrence sites) were recorded, with absence data lacking, which could introduce biases in model simulations. To enhance model accuracy, “pseudo‐absence points” were utilized (Inman et al. 2021). Given the high mobility of dholes, buffer zones with a radius twice the average daily movement distance were generated around the spatially thinned occurrence sites (Tananantayot et al. 2022). Background points were then randomly selected from areas outside these buffers, ensuring spatial independence (Barbet‐Massin et al. 2012; Gao et al. 2025). The accuracy of the distribution models was assessed using the True Skill Statistic (TSS) and the Area Under the Receiver Operating Characteristic Curve (AUC). Models with TSS and AUC values greater than 0.8 were considered highly accurate, and multiple models that met this criterion were selected to build an ensemble model (Gao et al. 2025). Seventy‐five percent of the samples were used as the training set, while the remaining 25% were reserved as the testing set, and 10 ensemble runs were conducted to enhance predictive reliability (Barbet‐Massin et al. 2012). The ensemble model predictions were imported into ArcGIS 10.8 for spatial analysis and visualization. The final model output was a probability raster (0–1000 scale), where values closer to 1000 indicated a higher likelihoods of dhole presence. For habitat classification, an optimal threshold (cut‐off) based on the TSS value was applied to divide the study area into suitable (values ≥ cut‐off) and unsuitable (values < cut‐off) zones (Gao et al. 2025).

### 2.3 Changes in Suitable Habitat

Changes in suitable habitats for dholes were analyzed by comparing habitat suitability values across past, current, and future periods. Suitable habitats were categorized into three types (Xue et al. 2021; Gao et al. 2025): (1) loss area—areas transitioning from suitable to unsuitable between periods; (2) unchanged area—areas consistently suitable across periods, serving as potential refugia; and (3) gain area—areas transitioning from unsuitable to suitable. The habitat change rate was calculated as $R_{c}=\left(S_{f}-S_{c}\right)/S_{c}\times 100\%$, where $S_{f}$ represents the suitable habitat area in the later period and $S_{c}$ represents that in the earlier period. Based on existing studies (Ding et al. 2024), we identified future refugia (unchanged area) as areas where habitats remain unchanged under future environmental changes. Furthermore, based on the home range of dholes (Sukmasuang et al. 2020), the core areas of future refugia (CFR) were defined as regions with an area greater than 133 km^2^.

### 2.4 Conservation Gaps

Conservation gap analysis identifies mismatches between a species' suitable habitat and existing protected areas, highlighting regions of high conservation value that are outside the current protection networks. These areas were overlaid with China's protected area layer (Zhao et al. 2024) to pinpoint regions that require protection but are currently outside conservation networks and to compare the differences in changes between suitable habitats and protected areas.

## 3 Results

### 3.1 Model Accuracy and Environmental Variable

**FIGURE 2:**
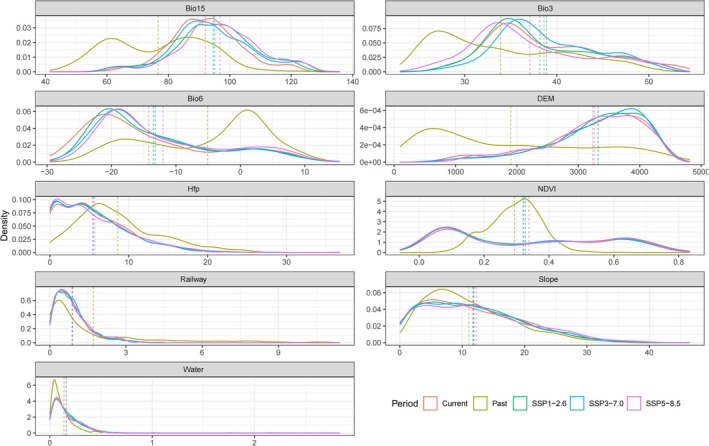
Kernel density distributions of environmental variables across past, current, and future climate periods.

The Biomod2 model for past/current scenarios achieved AUC values of 0.91 (past) and 0.95 (current), with corresponding TSS values of 0.84 and 0.90. The ranking of environmental variables importance varied (Table 1). In the past period, the top three important variables were the min temperature of coldest month (Bio6), DEM, and Slope, whereas in the current period, they are isothermality (Bio3), DEM, and slope. The kernel density distributions reveal distinct temporal shifts in the environmental drivers of *Cuon alpinus* habitat suitability (Figure 2). Suitable habitats have progressively shifted toward higher elevations and areas with greater isothermality (Bio3) values across historical, current, and future periods.

### 3.2 Changes in Suitable Habitat

**FIGURE 3:**
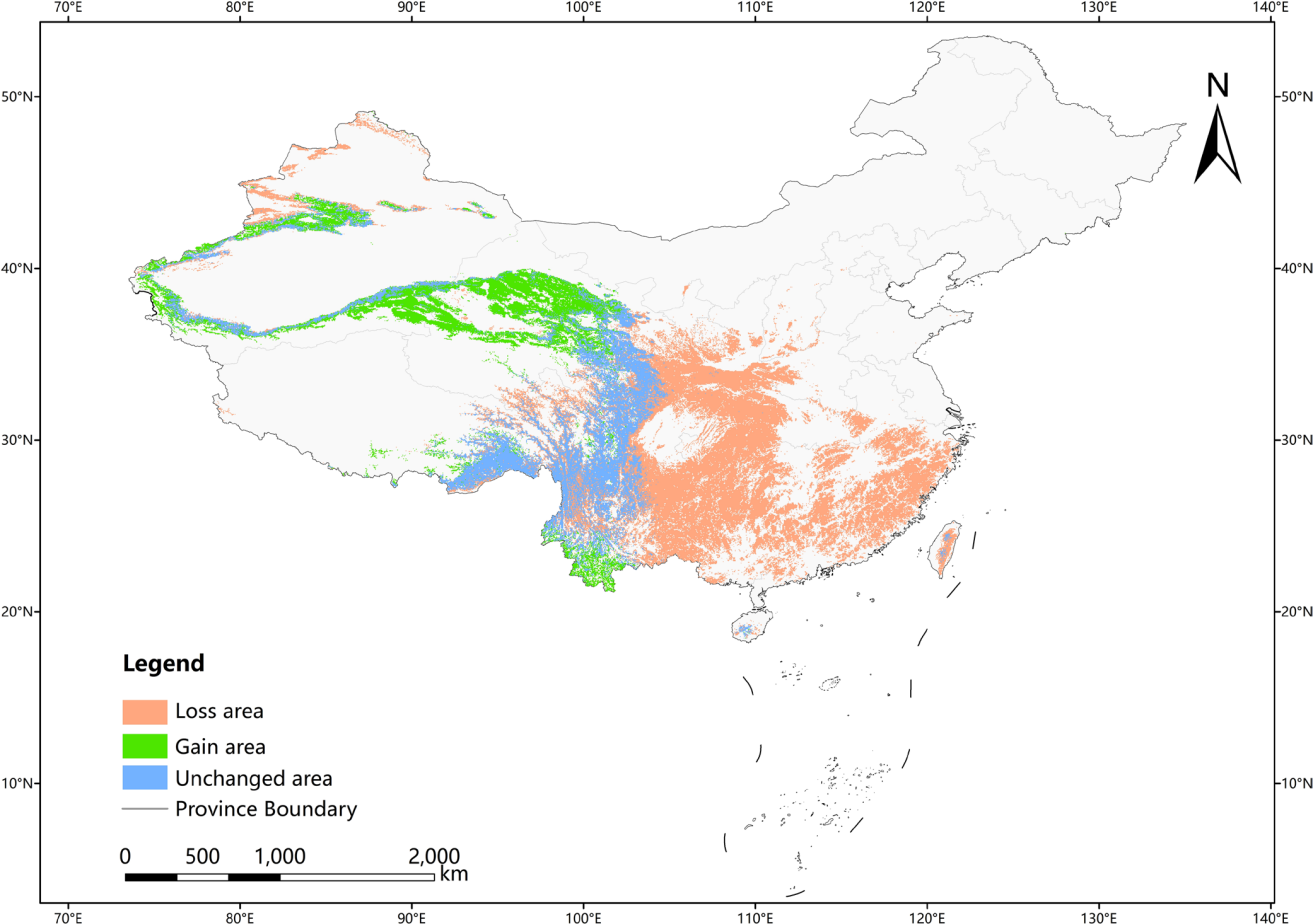
Assessment of dhole suitable habitats in past and current periods.

**FIGURE 4:**
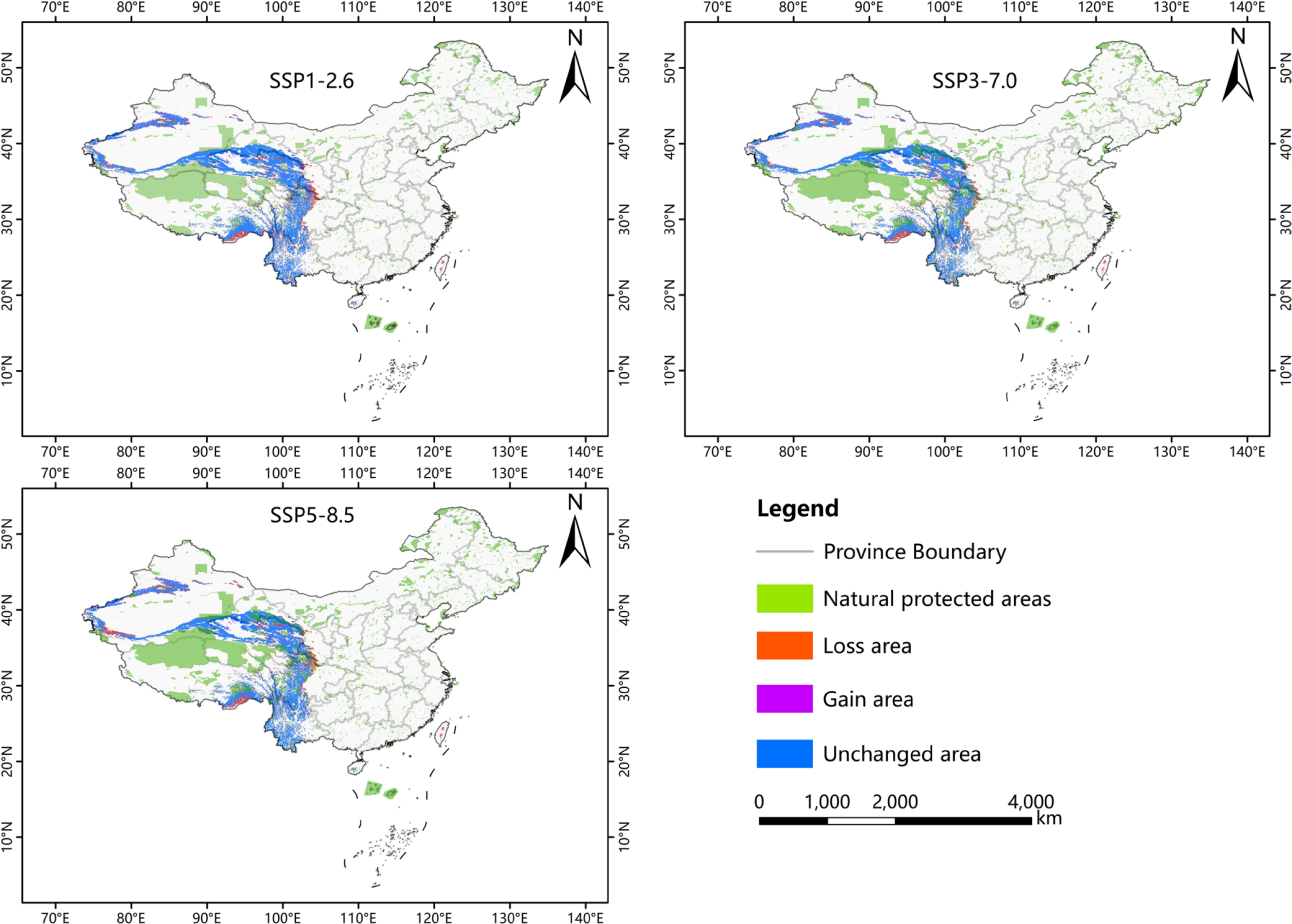
Assessment of dhole habitats under current and future conditions.

The current suitable habitat for dholes in China is estimated at 1,434,750 km^2^, primarily distributed around the Qinghai‐Tibet Plateau (Figure 3). Historically, suitable habitat for dholes spanned western, southern, and central China, covering an area of 2,737,625 km^2^. Currently, dholes have lost 1,897,700 km^2^ of habitat, gained 594,825 km^2^ of new habitat, and retained 839,925 km^2^ of stable habitat (Table 2). From 1980 to the present, the overall suitable habitat area has decreased by 47.59%. Under the future scenario, a decline of 12.04%–13.12% in suitable habitat for dholes is projected (Figure 4).

**TABLE 2:** Suitable habitat areas for dholes under different scenarios (km^2^).

Periods		Suitable area	Loss area	Gain area	Unchanged area	Range change (%)
past		2,737,625				
current		1,434,750	1,897,700	594,825	839,925	−47.59%
Future	SSP1‐2.6	1,262,000	209,400	36,650	1,225,350	−12.04%
	SSP3‐7.0	1,259,525	208,725	33,500	1,226,025	−12.21%
	SSP5‐8.5	1,246,525	226,650	38,425	1,208,100	−13.12%

### 3.3 Core Areas of Future Climate Refuge and Protection Gaps

**FIGURE 5:**
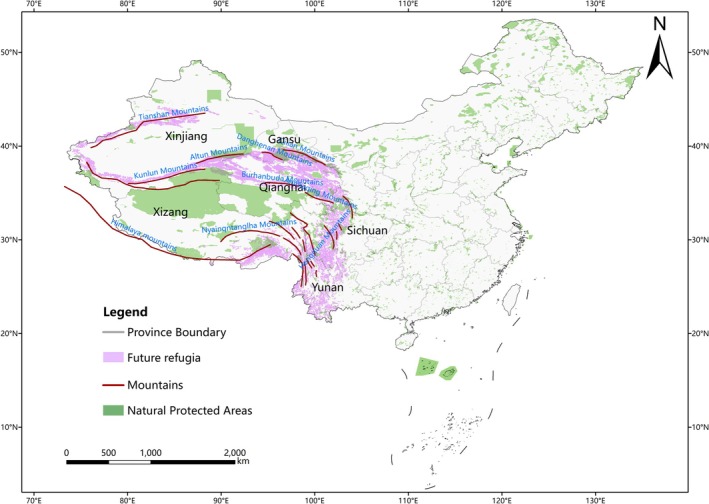
Distribution of future refugia for dholes.

Under climate change scenarios, the future refugia area for dholes is projected to range from 1,225,350 to 1,208,100 km^2^ (Table 2), with a core future refugia (CFR) area of 817,956 km^2^. These areas are distributed across six provincial‐level administrative regions: Xinjiang, Qinghai, Gansu, Tibet, Sichuan, and Yunnan. The primary CFR areas include the Kunlun‐Altun Mountains, Danghenan Mountains, Qilian Mountains, the southeast of Tibet, and the Hengduan Mountains (Figure 5).

However, an overlay analysis with China's protected areas highlights significant gaps in the protection of dhole habitats. Currently, only 242,975 km^2^ (16.94%) of suitable habitat is designated as protected, resulting in a substantial gap of 83.06%. Within the CFR, the proportion of unprotected areas is similarly high at 83.65%, though this varies among provinces; the largest gap is observed in Qinghai (90.57%), while Gansu shows the smallest gap at 57.71% (Table 3).

**TABLE 3:** The core areas of future refugia (CFR) for dholes (km^2^).

Province	CFR	CFR within protected areas	Gaps	Main distribution area
Gansu	81,459	34,447	57.71%	The Kunlun‐Altun Mountains and Tianshan Mountains
Qinghai	203,139	19,160	90.57%	North, central and east
Sichuan	139,232	22,658	83.73%	The Danghenan Mountains and Qilian Mountains
Tibet	88,767	14,176	84.03%	South and Southeast
Xinjiang	182,084	28,131	84.55%	The Hengduan Mountains
Yunnan	123,276	15,201	87.67%	North, West, and South
Total	817,956	133,773	83.65%	

## 4 Discussion

With the advancement of wildlife conservation research, numerous models have been developed to predict and evaluate species distributions and habitat dynamics. Since its release in 2003, the Biomod2 platform has been extensively applied across diverse taxa, including mammals (Biancolini et al. 2024), birds (Zhang et al. 2024), fish (Yang et al. 2022), and plants (Huang et al. 2023), to achieve robust assessments of species distribution ranges and habitats. The optimized ensemble model in our study consistently outperformed individual models across past and current periods. In the current period, its peak AUC (0.95) exceeded the best single‐model result (Random Forest: 0.94) by 1.06%, with similar trends in the past period. This highlights the ensemble's ability to reduce prediction variability while maintaining high accuracy, offering more reliable habitat distribution data for dholes.

### 4.1 Influence of Environmental Variables on Dhole Suitable Habitat

Dholes are habitat generalists, occupying a variety of ecosystems such as forests, grasslands, deserts, and alpine regions (Smith and Xie 2009; Thing et al. 2022; Srivathsa et al. 2023). In the Qilian Mountains of China, terrain ruggedness, livestock density, and mean annual temperature are key drivers of dhole habitat suitability (Liu, Wang, et al. 2024), while Yang et al. (2025) highlight mean diurnal range (Bio2), temperature seasonality (Bio4), and elevation as critical factors in MaxEnt‐based simulations. In Southeast Asia, forest cover and prey availability dominate habitat suitability (Tananantayot et al. 2022), whereas precipitation of driest quarter (Bio17) and land‐use/land‐cover were most influential in India's Pench Tiger Reserve (Jain et al. 2020). These pronounced regional and methodological variations underscore the context‐dependent nature of habitat modeling and highlight a critical gap: the lack of a consolidated understanding of the primary drivers shaping dhole distribution at a broader, national scale. Addressing this gap, our analysis for China identified isothermality (Bio3), elevation, and slope as the predominant drivers of current dhole habitat. Bio3, reflecting temperature stability, indicates that dholes are sensitive to thermal variability, a finding consistent with observed seasonal activity shifts along the northeastern Qinghai‐Tibet Plateau (Huang et al. 2025). Furthermore, elevation and slope, as fundamental topographic factors, indirectly shape habitat suitability by governing temperature regimes, precipitation patterns, vegetation types, and oxygen levels (Li et al. 2021).

### 4.2 Past Versus Current Suitable Habitat

Historically, dholes were widely distributed across China, occupying all regions except Hainan and Taiwan (Smith and Xie 2009; Liu et al. 2019). However, since the early 2000s, confirmed records of dhole presence have become increasingly scarce, now limited to provinces such as Xinjiang, Gansu, Qinghai, Shaanxi, Sichuan, Yunnan, and Tibet (Liu et al. 2019). Our analysis indicates a dramatic contraction of suitable habitat compared to historical ranges. For instance, in Sichuan Province alone, dhole habitat has diminished by 75.65% (Xia et al. 2023), becoming restricted to fragmented patches within western protected areas (Liu, Ji, et al. 2024). Nationally, the distribution of dholes has declined by approximately 50% between past and present periods, with lost habitats primarily concentrated in southeastern China (Figure 2). This significant range contraction appears to be driven by a complex interplay of climatic, topographic, and anthropogenic factors.

The model reveals that Bio6 historically served as a significant explanatory factor for the spatial pattern of dhole suitable habitat distribution. This finding is corroborated by its contribution rate of 58.95% (Table 1) in species distribution modeling, confirming that low‐temperature conditions have been a critical environmental threshold limiting dhole distribution in the past. However, in the current period, the model contribution rate for Bio6 has decreased to 4.59%. This sharp decline indicates that temperature constraints have diminished in modern times, no longer serving as a dominant force shaping dhole distribution patterns. Conversely, the importance of Bio3 has increased, making it the primary explanatory factor, with its contribution rate increasing by 125%. This shift suggests that as macro‐temperature constraints weaken, the stability of temperature variation—reflected in diurnal and seasonal temperature differences—has become a more critical climate‐selective factor. In the current period, the model contribution rate for DEM has risen from 41.02% to 54.71%, corroborating that suitable habitat for dholes has greatly contracted and clustered in specific high‐altitude areas. The refuge effect provided by topography may be crucial for resisting human disturbances and climate change. Although the contribution rate for Slope has climbed from 6.65% to 10.48%, this implies that dholes' suitable habitat will be more prone to being fragmented into isolated patches.

The impact of human activities is complex. The contribution rate for Hfp decreased from 4.72% to 0.83% exhibited a downward trends. This decline reflects not a weakening of human impact, but a narrowing contrast between presence and pseudo‐absence points as the background human footprint has risen. In the past, when footprint values were generally low, presence points in undisturbed areas and pseudo‐absence points spanning wider disturbance levels exhibited a clear contrast. As the landscape‐wide footprint has since increased substantially, values at both point types have risen, shrinking their relative difference and reducing Hfp's discriminatory power. Conversely, the contribution rate for Railway (from 1.73% to 2.25%) has shown slight increases, indicating that the barrier and avoidance effects of linear infrastructure have become more pronounced in local contexts.

Additionally, some researchers hypothesize that the rapid disappearance of suitable dhole habitats in southeastern China is primarily driven by factors such as diminished prey resources, the spillover of diseases transmitted by domestic dogs, and intensified conflicts between humans and dholes (Kamler et al. 2015; Tananantayot et al. 2022; Xia et al. 2023). While these drivers are strongly implicated in correlative studies, their relative contributions and precise mechanisms were not directly measured or modeled in this research. Future studies integrating direct empirical data are necessary to quantitatively test these hypotheses. Clearly, the fundamental shift in these driving factors serves as the underlying mechanism leading to the dramatic compression of dhole distribution and its concentration in western mountainous regions.

### 4.3 Future Versus Current Suitable Habitat

Climate change collectively threaten global biodiversity by inducing habitat degradation, genetic diversity erosion, altered species interactions, proliferation of invasive species, and intensified human‐wildlife conflicts (Bellard et al. 2012; Scheffers et al. 2016; Ma et al. 2024). As a component of this global trend, dholes face escalating pressures driven by these anthropogenic environmental changes (de Oliveira et al. 2023). Our future simulations across three distinct scenarios (SSP1‐2.6, SSP3‐7.0, and SSP5‐8.5) consistently project a declining trend in suitable habitat for dholes, underscoring the significant threats posed by impending climate shifts. The current suitable habitat, with an average elevation of 3262 ± 883 m, aligns with the documented preference of dholes for mid‐to‐high altitude zones (Yang et al. 2025; Namgyal and Thinley 2017). These landscapes provide dual advantages: they support robust populations of key prey species, such as Blue Sheep ( *Pseudois nayaur* ), Muntjacs (*Muntiacus vaginalis*), and Tibetan Antelope ( *Pantholops hodgsonii* ) (Cong et al. 2024; Shi et al. 2023), and offer rugged terrain rich in microhabitats that are crucial for shelter, breeding, and hunting strategies.

Future projections, however, suggest that dhole distribution will be characterized by a contraction at the western, eastern, and southwestern edges of the suitable habitat, alongside an upward elevational shift toward regions with a marginally higher mean elevation (3283 ± 850 m) (Figure 2). This upward shift is more indicative of a range contraction into fragmented high‐elevation refugia rather than a uniform, upward migration of the entire habitat. This spatial dynamic highlights the pivotal role of topography—encompassing elevation, slope, and ruggedness—in mediating the impacts of climate change on dhole distribution across China (Yang et al. 2025; Huang et al. 2025; Liu, Wang, et al. 2024; Xia et al. 2023).

In addition, the recent increase in dhole sightings in South Asia (Kazi et al. 2021; Vaghela 2024; Ghimirey et al. 2024; Nguyen et al. 2026) suggests that similar records are likely to emerge in China. Supporting evidence already exists: scientific records from high‐altitude mountain and plateau regions such as the Altun Mountains of Xinjiang (Xue et al. 2014) and our new finding in Delingha (Table S1), Qinghai, along with credible recent media reports confirming a sighting via infrared camera in the Taxkorgan Nature Reserve in Xinjiang's Pamir region—an area where dholes were historically present (Xiao et al. 2021). Given the species' once widespread distribution in China, its high dispersal ability, and the strengthening of wildlife protection measures, an increase in dhole records across China is therefore anticipated in the coming years.

Consequently, these findings underscore the critical role of topography, particularly high‐elevation refugia, in mediating the impacts of climate change on dhole persistence in China. Protecting these refugia and ensuring landscape connectivity will be paramount for the species' long‐term conservation under future climate scenarios.

### 4.4 Core Suitable Habitat of Dholes in the Future Under Climate Change

Conservation gap analysis indicates that the protection gap for current and future suitable habitats of Chinese dholes exceeds 80% (Figure 4), highlighting the urgent need to strengthen conservation efforts further and integrate additional suitable habitats into protected areas. China's ongoing initiatives to enhance its protected area system—particularly through the establishment of a national park network—promise to improved habitat coverage and climate connectivity (Wang and Gao 2024; Xu et al. 2024). Key regions include the Kunlun‐Altun Mountains, Danghenan Mountains, Qilian Mountains, southeastern Tibet, and the Hengduan Mountains (Figure 5). These areas provide extensive habitats for dholes, with numerous sightings documented, and are also identified as CFRs for dholes in the context of climate change. Among the proposed national park candidates (Tang et al. 2023), several sites overlap with dhole CFRs, including the Kunlun Mountains, Qilian Mountains, Yarlung Tsangpo Grand Canyon, Gongga Mountain, Shangri‐La, Gaoligong Mountains, Ailao Mountains, and habitats for the Asian Elephant. Systematic protection measures (e.g., establishing protected areas and management plans, conducting continuous monitoring, restoring ungulate populations, and alleviating human‐dhole conflicts) could significantly enhance conservation outcomes for dholes, thereby mitigating threats posed by climate change and human activities.

### 4.5 Study Limitations

While this study offers valuable insights into the dynamics of dhole habitats—past, current, and future—several limitations must be acknowledged: (1) The past occurrence records were sourced from literature, which complicates the verification of their accuracy or spatial precision. (2) The scarcity of occurrence data may undermine the robustness of species distribution models, particularly in regions that have not been extensively surveyed. (3) The specific mechanisms through which climate change affect dhole habitats—especially at fine spatial scales or within homogeneous ecosystems—require further investigation. Addressing these limitations through improved field surveys, interdisciplinary research, and mechanistic modeling will enhance future conservation strategies.

## 5 Conclusions

This study integrated historical and contemporary occurrence records of the dhole ( *Cuon alpinus* ) with projected climate change scenarios to analyze spatiotemporal habitat dynamics across China using ensemble species distribution models. The results indicate that suitable habitat is predominantly located along the periphery of the Qinghai‐Tibet Plateau. From 1980 to the present, the total area of suitable habitat has contracted by 47.59%. Under the climate change scenarios, models project further habitat reductions of 12.04%–13.12%. The identified core areas for future refugia include the Kunlun‐Altun Mountains, Danghenan Mountains, Qilian Mountains, southeastern Tibet, and Hengduan Mountains. Despite advancements in conservation efforts, significant habitat deficits persist, amounting to nearly half of the past range. This necessitates urgent expansion of protected area networks and evidence‐based adaptive management in core zones. These findings underscore the impact of climate change on dhole persistence in China, highlighting the urgent need for spatially explicit, climate‐adaptive conservation strategies.

## Author Contributions

**Taifu Huang:** data curation (equal), methodology (equal), writing – original draft (equal), writing – review and editing (equal). **Shuaishuai Gao:** methodology (equal), software (equal). **Wei Cong:** data curation (equal). **Jianhua Yu:** data curation (equal). **Zhen Li:** data curation (equal). **Yuan Yang:** data curation (equal). **Tong Zhang:** data curation (equal). **Yuan Wang:** data curation (equal). **Zhonghua Wang:** data curation (equal). **Jia Li:** data curation (equal), writing – original draft (equal). **Yadong Xue:** data curation (equal), resources (equal). **Yuguang Zhang:** data curation (equal), funding acquisition (equal), project administration (equal), writing – original draft (equal), writing – review and editing (equal).

## Funding

This research was funded by The Third Xinjiang Scientific Expedition Program (Grant No. 2021xjkk1203) and National Natural Science Foundation of China (Grant No. 32201430).

## Conflicts of Interest

The authors declare no conflicts of interest.

## Supporting information


**Figure S1:** Analysis of correlation coefficients of the environmental variables.
**Table S1:** Sources of dholes occurrence.
**Table S2:** Model parameters by algorithm.

## Data Availability

All the required data are uploaded as Supporting Information, with the exception of protected area data. Due to management requirements regarding the boundary scope of the protected area, this data cannot be disclosed.

## References

[ece373822-bib-0002] Bellard, C. , C. Bertelsmeier , P. Leadley , W. Thuiller , and F. Courchamp . 2012. “Impacts of Climate Change on the Future of Biodiversity.” Ecology Letters 15, no. 4: 365–377. 10.1111/j.1461-0248.2011.01736.x. 22257223 PMC3880584

[ece373822-bib-0003] Bennington, S. , P. W. Dillingham , S. D. Bourke , S. M. Dawson , E. Slooten , and W. J. Rayment . 2024. “Testing Spatial Transferability of Species Distribution Models Reveals Differing Habitat Preferences for an Endangered Delphinid ( *Cephalorhynchus hectori* ) in Aotearoa, New Zealand.” Ecology and Evolution 14, no. 7: e70074. 10.1002/ece3.70074. 39041012 PMC11262828

[ece373822-bib-0004] Bestion, E. , A. Soriano‐Redondo , J. Cucherousset , et al. 2019. “Altered Trophic Interactions in Warming Climates: Consequences for Predator Diet Breadth and Fitness.” Proceedings of the Royal Society B: Biological Sciences 286, no. 1914: 20192227. 10.1098/rspb.2019.2227. PMC6834436 31662087

[ece373822-bib-0005] Biancolini, D. , M. Pacifici , M. Falaschi , et al. 2024. “Global Distribution of Alien Mammals Under Climate Change.” Global Change Biology 30, no. 11: e17560. 10.1111/gcb.17560. 39545282

[ece373822-bib-0006] Calhoun, K. L. , J. A. Smith , M. W. Tingley , et al. 2025. “Human‐Wildlife Conflict Is Amplified During Periods of Drought.” Science Advances 11, no. 46: eadx0286. 10.1126/sciadv.adx0286. 41223262 PMC12609059

[ece373822-bib-0007] Charaspet, K. , R. Sukmasuang , N. Khoewsree , M. Pla‐ard , and Y. Chanachai . 2020. “Prey Species and Prey Selection of Dholes at Three Different Sites in Thailand.” Biodiversitas 21, no. 11: 5248–5262. 10.13057/biodiv/d211128.

[ece373822-bib-0009] Chen, Z. Q. , B. L. Yu , C. S. Yang , et al. 2021. “An Extended Time‐Series (2000–2018) of Global NPP‐VIIRS‐Like Nighttime Light Data From a Cross‐Sensor Calibration.” Earth System Science Data Discussions 13, no. 3: 889–906. 10.5194/essd-13-889-2021.

[ece373822-bib-0011] Choki, K. , P. Dhendup , J. Tenzin , et al. 2023. “Conservation Potential of Non‐Protected Area for Sympatric Carnivores in Bhutan.” Global Ecology and Conservation 42: e02392. 10.1016/j.gecco.2023.e02392.

[ece373822-bib-0012] Cohen, J. M. , and W. Jetz . 2025. “Geographic Redistributions Are Insufficient to Mitigate Exposure to Climate Change in North American Birds.” Nature Ecology & Evolution 9, no. 7: 1234–1244. 10.1038/s41559-025-02714-7. 40437204

[ece373822-bib-0013] Cong, W. , Y. Zhang , T. F. Huang , et al. 2024. “Dietary Composition and Niche Partitioning of Sympatric Carnivores in Altun Mountain National Nature Reserve.” Acta Theriologica Sinica 44, no. 6: 695–705. (in Chinese). 10.16829/j.slxb.150941.

[ece373822-bib-0014] de Oliveira, G. L. , A. B. Viana‐Junior , P. H. S. Trindade , et al. 2023. “Wild Canids and the Ecological Traps Facing the Climate Change and Deforestation in the Amazon Forest.” Ecology and Evolution 13, no. 6: e10150. 10.1002/ece3.10150. 37304361 PMC10251424

[ece373822-bib-0015] Ding, J. J. , Y. Y. Zhuo , W. X. Xu , M. Kessler , M. Y. Wang , and W. K. Yang . 2024. “Synergistic Effects of Climate and Land Use Change on Khulan ( *Equus hemionus hemionus* ) Habitat in China.” Global Ecology and Conservation 54: e03181. 10.1016/j.gecco.2024.e03181.

[ece373822-bib-0016] Elith, J. , and J. R. Leathwick . 2009. “Species Distribution Models: Ecological Explanation and Prediction Across Space and Time.” Annual Review of Ecology, Evolution, and Systematics 40: 677–697. 10.1146/annurev.ecolsys.110308.120159.

[ece373822-bib-0017] Gao, S. S. , D. Davaasuren , J. C. Li , et al. 2025. “Projected Impacts of Climate Change and Border Fencing on the Distribution of Khulan ( *Equus hemionus hemionus* ).” Global Ecology and Conservation 60: e03593. 10.1016/j.gecco.2025.e03593.

[ece373822-bib-0019] Ghaskadbi, P. , P. Nigam , and B. Habib . 2022. “Stranger Danger: Differential Response to Strangers and Neighbors by a Social Carnivore, the Asiatic Wild Dog ( *Cuon alpinus* ).” Behavioral Ecology and Sociobiology 76, no. 7: 86. 10.1007/s00265-022-03188-4.

[ece373822-bib-0020] Ghimirey, Y. , R. Acharya , K. Yadav , et al. 2024. “Challenges and Possible Conservation Implications of Recolonizing Dholes *Cuon alpinus* in Nepal.” Oryx 58, no. 3: 378–386. 10.1017/s003060532300073x.

[ece373822-bib-0021] Guo, J. H. , H. T. Jia , Y. X. Wang , X. Wang , and W. Li . 2024. “Dynamical Downscaling of Daily Extreme Temperatures Over China Using PRECIS Model.” Sustainability 16, no. 7: 3030. 10.3390/su16073030.

[ece373822-bib-0022] Hao, T. X. , J. Elith , G. Guillera‐Arroita , and J. J. Lahoz‐Monfort . 2019. “A Review of Evidence About Use and Performance of Species Distribution Modelling Ensembles Like BIOMOD.” Diversity and Distributions 25, no. 5: 839–852. 10.1111/ddi.12892.

[ece373822-bib-0023] Hao, T. X. , J. Elith , J. J. Lahoz‐Monfort , and G. Guillera‐Arroita . 2020. “Testing Whether Ensemble Modelling Is Advantageous for Maximising Predictive Performance of Species Distribution Models.” Ecography 43, no. 4: 549–558. 10.1111/ecog.04890.

[ece373822-bib-0024] Hellegers, M. , W. A. Ozinga , A. van Hinsberg , et al. 2020. “Evaluating the Ecological Realism of Plant Species Distribution Models With Ecological indicator Values.” Ecography 43: 161–170. 10.1111/ecog.04291.

[ece373822-bib-0025] Hoffmann, S. , S. D. H. Irl , and C. Beierkuhnlein . 2019. “Predicted Climate Shifts Within Terrestrial Protected Areas Worldwide.” Nature Communications 10, no. 1: 4787. 10.1038/s41467-019-12603-w. PMC6803628 31636257

[ece373822-bib-0027] Huang, D. Y. , Q. J. An , S. P. Huang , et al. 2023. “Biomod2 Modeling for Predicting the Potential Ecological Distribution of Three Fritillaria Species Under Climate Change.” Scientific Reports 13, no. 1: 18801. 10.1038/s41598-023-45887-6. 37914761 PMC10620159

[ece373822-bib-0028] Huang, T. F. , Y. Zhang , X. M. Liu , et al. 2025. “Spatial and Temporal Relationships Among Wolf ( *Canis lupus* ), dhole ( *Cuon alpinus* ), and Red Fox ( *Vulpes vulpes* ).” Acta Ecologica Sinica 45, no. 13: 6600–6608. (in Chinese). 10.20103/j.stxb.202407151650.

[ece373822-bib-0029] Inman, R. , J. Franklin , T. Esque , and K. Nussear . 2021. “Comparing Sample bias Correction Methods for Species Distribution Modeling Using Virtual Species.” Ecosphere 12, no. 3: e03422. 10.1002/ecs2.3422.

[ece373822-bib-0030] Jain, D. , G. Areendran , K. Raj , V. D. Gupta , and M. Sahana . 2020. “Comparison of AHP and Maxent Model for Assessing Habitat Suitability of Wild Dog ( *Cuon alpinus* ) in Pench Tiger Reserve, Madhya Pradesh.” In Spatial Modeling in Forest Resources Management: Rural Livelihood and Sustainable Development, edited by P. K. Shit , H. R. Pourghasemi , P. Das , and G. S. Bhunia , 327–363. Springer International Publishing. 10.1007/978-3-030-56542-8_14.

[ece373822-bib-0031] Kamler, J. F. , N. Songsasen , K. Jenks , et al. 2015. *Cuon alpinus* . The IUCN Red List of Threatened Species 2015: e.T5953A72477893. 10.2305/IUCN.UK.2015-4.RLTS.T5953A72477893.en.

[ece373822-bib-0032] Kazi, A. A. , D. N. Rabari , M. I. Dahya , and S. Lyngdoh . 2021. “Reappearance of Dhole *Cuon alpinus* (Mammalia: Carnivora: Canidae) in Gujarat After 70 Years.” Journal of Threatened Taxa 13, no. 6: 18655–18659. 10.11609/jott.6415.13.6.18655-18659.

[ece373822-bib-0033] Lenoir, J. , J. C. Gégout , P. A. Marquet , P. de Ruffray , and H. Brisse . 2008. “A Significant Upward Shift in Plant Species Optimum Elevation During the 20th Century.” Science 320, no. 5884: 1768–1771. 10.1126/science.1156831. 18583610

[ece373822-bib-0035] Li, Z. L. , L. A. Duo , S. Li , and T. M. Wang . 2021. “Competition and Coexistence Among Terrestrial Mammalian Carnivores.” Biodiversity Science 29, no. 1: 81–97. (in Chinese). 10.17520/biods.2020359.

[ece373822-bib-0036] Liu, H. , Q. Liu , X. Cui , et al. 2025. “Prediction of Potential Suitable Habitats for *Elaphodus cephalophus* in China Under Climate Change Scenarios.” Ecology and Evolution 15, no. 10: e72194. 10.1002/ece3.72194. 41019403 PMC12463574

[ece373822-bib-0037] Liu, K. , S. N. Ji , T. P. Guan , and S. Li . 2024. “A Preliminary Study of the Historical and Current Distribution of Dhole ( *Cuon alpinus* ) in Sichuan Province.” Acta Theriologica Sinica 44, no. 6: 804–814. (in Chinese). 10.16829/j.slxb.150981.

[ece373822-bib-0038] Liu, S. Y. , Y. Wu , and S. Li . 2019. Handbook of the Mammals of China. 2nd ed. Straits Book Company (in Chinese).

[ece373822-bib-0039] Liu, Y. L. , Y. D. Wang , Y. B. Li , et al. 2024. “The Current Distribution and Prediction of Suitable Habitat of Dhole ( *Cuon alpinus* ) in Qilian Mountains, China.” Acta Theriologica Sinica 44, no. 6: 749–761. (in Chinese). 10.16829/j.slxb.150969.

[ece373822-bib-0040] Luo, M. , H. Wang , and Z. Lyu . 2017. “Evaluating the Performance of Species Distribution Models Biomod2 and MaxEnt Using the Giant Panda Distribution Data.” Chinese Journal of Applied Ecology 28, no. 12: 4001–4006. (in Chinese). 10.13287/j.1001-9332.201712.011. 29696896

[ece373822-bib-0041] Ma, D. Q. , B. Abrahms , J. Allgeier , T. Newbold , B. C. Weeks , and N. H. Carter . 2024. “Global Expansion of Human‐Wildlife Overlap in the 21st Century.” Science Advances 10, no. 34: eadp7706. 10.1126/sciadv.adp7706. 39167651 PMC11338222

[ece373822-bib-0042] Ma, F. , and J. L. Zhang . 2009. Survey on Key Terrestrial Wildlife Resources in China. China Forestry Publishing House.

[ece373822-bib-0043] Ma, Z. B. , P. Wang , Y. B. Li , et al. 2023. “Population Survey for *Cuon alpinus* in the Yanchiwan National Nature Reserve of Gansu.” Journal of Gansu Forestry Science and Technolgy 48, no. 3: 57–59. 10.3969/j.issn.1006-0960.2023.03.012.

[ece373822-bib-0044] Marciszak, A. , A. Kropczyk , W. Gornig , M. Kot , A. Nadachowski , and G. Lipecki . 2023. “History of Polish Canidae (Carnivora, Mammalia) and Their Biochronological Implications on the Eurasian Background.” Genes 14, no. 3: 539. 10.3390/genes14030539. 36980812 PMC10048199

[ece373822-bib-0045] Marmion, M. , M. Parviainen , M. Luoto , R. K. Heikkinen , and W. Thuiller . 2009. “Evaluation of Consensus Methods in Predictive Species Distribution Modelling.” Diversity and Distributions 15, no. 1: 59–69. 10.1111/j.1472-4642.2008.00491.x.

[ece373822-bib-0046] Mu, H. W. , X. C. Li , Y. N. Wen , et al. 2022. “A Global Record of Annual Terrestrial Human Footprint Dataset From 2000 to 2018.” Scientific Data 9: 176. 10.1038/s41597-022-01284-8. 35440581 PMC9018937

[ece373822-bib-0090] Namgyal, C. , and P. Thinley . 2017. “Distribution and Habitat Use of the Endangered Dhole *Cuon alpinus* (Pallas, 1811) (Mammalia: Canidae) in Jigme Dorji National Park, Western Bhutan.” Journal of Threatened Taxa 9, no. 9: 10649. 10.11609/jott.3091.9.9.10649-10655.

[ece373822-bib-0047] Nguyen, A. T. , A. Nguyen , T. V. Nguyen , et al. 2026. “New Record of Dhole *Cuon alpinus* in Vietnam, Yet Apparent Decline of the Species Across the Country.” Oryx: 1–4. 10.1017/S0030605325101580.

[ece373822-bib-0048] Nogués‐Bravo, D. , F. Rodríguez‐Sánchez , L. Orsini , et al. 2018. “Cracking the Code of Biodiversity Responses to Past Climate Change.” Trends in Ecology & Evolution 33, no. 10: 765–776. 10.1016/j.tree.2018.07.005. 30173951

[ece373822-bib-0049] Popovici, D. C. , O. Ionescu , G. Ionescu , et al. 2025. “Two Decades of Expansion: Population Dynamics and Spatial Distribution of the Golden Jackal in Romania Between 2004–2025.” Bulletin of the Transilvania University of Brasov. Series II: Forestry• Wood Industry• Agricultural Food Engineering 18, no. 1: 41–54. 10.31926/but.fwiafe.2025.18.67.1.3.

[ece373822-bib-0050] Porto, L. M. V. , D. Bennett , R. Maestri , and R. S. Etienne . 2024. “Canids in a Changing Climate: Predictingrange Shifts and Evolutionary Rescue Under Distinct Future Scenarios.” Biological Journal of the LinneanSociety 143, no. 2: blae094. 10.1093/biolinnean/blae094.

[ece373822-bib-0051] Punjabi, G. A. , L. W. Havmøller , R. W. Havmøller , D. Ngoprasert , and A. Srivathsa . 2022. “Methodological Approaches for Estimating Populations of the Endangered Dhole *Cuon alpinus* .” PeerJ 10: e12905. 10.7717/peerj.12905. 35223205 PMC8877337

[ece373822-bib-0052] Risely, A. , N. Müller‐Klein , D. W. Schmid , et al. 2023. “Climate Change Drives Loss of Bacterial Gut Mutualists at the Expense of Host Survival in Wild Meerkats.” Global Change Biology 29, no. 20: 5816–5828. 10.1111/gcb.16877. 37485753

[ece373822-bib-0053] Scheffers, B. R. , L. De Meester , T. C. Bridge , et al. 2016. “The Broad Footprint of Climate Change From Genes to Biomes to People.” Science 354, no. 6313: aaf7671. 10.1126/science.aaf7671. 27846577

[ece373822-bib-0056] Shi, X. Y. , X. Y. Li , C. Y. Wei , et al. 2023. “Avian and Mammal Diversities and Their Altitudinal and Seasonal Distribution Patterns in Yarlung Zangbo Grand Canyon, China.” Biodiversity Science 31: 22491. (in Chinese). 10.17520/biods.2022491.

[ece373822-bib-0057] Smith, A. T. , and Y. Xie . 2009. A Guide to the Mammals of China. Hunan Education Publishing House (in Chinese).

[ece373822-bib-0059] Srivathsa, A. , V. Ramachandran , P. Saravanan , A. Sureshbabu , D. Ganguly , and U. Ramakrishnan . 2023. “Topcats and Underdogs: Intraguild Interactions Among Three Apex Carnivores Across Asia's Forestscapes.” Biological Reviews 98, no. 6: 2114–2135. 10.1111/brv.12998. 37449566

[ece373822-bib-0061] Sukmasuang, R. , W. Suksavate , N. Songsasen , et al. 2020. “Home Range, Movement and Habitat Selection of Dholes ( *Cuon alpinus* ) in Khao Yai National Park, Thailand.” Biodiversitas 21, no. 12: 5915–5926. 10.13057/biodiv/d211257.

[ece373822-bib-0062] Sunday, J. M. 2020. “The Pace of Biodiversity Change in a Warming Climate.” Nature 580, no. 7804: 460–461. 10.1038/d41586-020-00975-9. 32269374

[ece373822-bib-0063] Tananantayot, J. , C. Agger , E. Ash , et al. 2022. “Where Will the Dhole Survive in 2030? Predicted Strongholds in Mainland Southeast Asia.” Conservation Science and Practice 4, no. 11: e12831. 10.1111/csp2.12831.

[ece373822-bib-0064] Tang, X. P. , Z. Y. Ouyang , Y. F. Jiang , et al. 2023. “Spatial Planning of National Parks in China.” National Park 1, no. 1: 1–10. (in Chinese). 10.20152/j.np.2023.01.001.

[ece373822-bib-0065] Thing, S. B. , J. B. Karki , B. R. Lamichhane , S. Shrestha , U. R. Regmi , and R. Ranabhat . 2022. “Distribution and Habitat‐Use of Dhole Cuon alpinus (Mammalia: Carnivora: Canidae) in Parsa National Park, Nepal.” Journal of Threatened Taxa 14, no. 3: 20703–20712. 10.11609/jott.6178.14.3.20703-20712.

[ece373822-bib-0066] Thinley, P. , R. Rajaratnam , J. F. Kamler , and C. Wangmo . 2021. “Conserving an Endangered Canid: Assessing Distribution, Habitat Protection, and Connectivity for the Dhole ( *Cuon alpinus* ) in Bhutan.” Frontiers in Conservation Science 2: 654976. 10.3389/fcosc.2021.654976.

[ece373822-bib-0067] Thompson, M. S. A. , E. Couce , M. Schratzberger , and C. P. Lynam . 2023. “Climate Change Affects the Distribution of Diversity Across Marine Food Webs.” Global Change Biology 29, no. 23: 6606–6619. 10.1111/gcb.16881. 37814904 PMC10946503

[ece373822-bib-0068] Thuiller, W. , B. Lafourcade , R. Engler , and M. B. Araújo . 2009. “Biomod: A Platform for Ensemble Forecasting of Species Distributions.” Ecography 32: 369–373. 10.1111/j.1600-0587.2008.05742.x.

[ece373822-bib-0070] Vaghela, U. 2024. “Dholes in the Vicinity of Bhimashankar Wildlife Sanctuary, Maharashtra After 189 Years: First Photographic Record.” Zoo's Print 39, no. 8: 32–33.

[ece373822-bib-0072] Wang, W. , and Z. X. Gao . 2024. “Progress and Prospects of the Construction of Protected Area System With National Parks as the Main Body in China. Research of.” Environmental Sciences 37, no. 10: 2100–2109. (in Chinese). 10.13198/j.issn.1001-6929.2024.09.08.

[ece373822-bib-0075] Wolf, C. , and W. J. Ripple . 2017. “Range Contractions of the World's Large Carnivores.” Royal Society Open Science 4: 170052. 10.1098/rsos.170052. 28791136 PMC5541531

[ece373822-bib-0076] Wu, X. Y. , Q. G. Wei , D. Sai , and H. Zhang . 2021. “China's Dhole Population at Risk of Extinction.” Science 372, no. 6541: 472. 10.1126/science.abi8889. 33926944

[ece373822-bib-0078] Xia, W. C. , C. C. Grueter , C. Zhang , et al. 2023. “Distribution Range Contractions and Identification of Conservation Priority Areas for Canids in Sichuan Province, China.” Global Ecology and Conservation 44: e02499. 10.1016/j.gecco.2023.e02499.

[ece373822-bib-0091] Xiao, Y. , X. B. Cai , X. Y. Zhang , et al. 2021. “Investigation of Avian and Mammalian Resources in the Taxkorgan Wildlife Nature Reserve, Xinjiang.” Sichuan Journal of Zoology 40, no. 1: 75–85. (in Chinese). 10.11984/j.issn.1000-7083.20200180.

[ece373822-bib-0079] Xu, D. M. , J. Peng , J. Q. Dong , et al. 2024. “Expanding China's Protected Areas Network to Enhance Resilience of Climate Connectivity.” Science Bulletin 69, no. 14: 2273–2280. 10.1016/j.scib.2024.04.036. 38724302

[ece373822-bib-0080] Xue, Y. D. , J. Li , Y. Zhang , et al. 2021. “Assessing the Vulnerability and Adaptation Strategies of Wild Camel to Climate Change in the Kumtag Desert of China.” Global Ecology and Conservation 29: e01725. 10.1016/j.gecco.2021.e01725.

[ece373822-bib-0081] Xue, Y. D. , F. Liu , T. Z. Guo , L. Yuan , and D. Q. Li . 2014. “Using Camera Traps to Survey Wildlife at Water Sources on the Northern Slope of the Altun Mountains, China.” Acta Theriologica Sinica 34, no. 2: 164–171. (in Chinese). 10.16829/j.slxb.2014.02.008.

[ece373822-bib-0082] Yang, T. Y. , X. Y. Liu , and Z. Q. Han . 2022. “Predicting the Effects of Climate Change on the Suitable Habitat of Japanese Spanish Mackerel ( *Scomberomorus niphonius* ) Based on the Species Distribution Model.” Frontiers in Marine Science 9: 927790. 10.3389/fmars.2022.927790.

[ece373822-bib-0083] Yang, Y. G. , P. Luo , Y. Zhao , T. Z. Zhang , F. Jiang , and Z. Q. You . 2025. “Distribution Patterns and Ecological Determinants of Suitable Habitats for the Dhole ( *Cuon alpinus* ) in China.” Animals 15, no. 4: 463. 10.3390/ani15040463. 40002945 PMC11852048

[ece373822-bib-0086] Zhang, X. , L. Liu , X. Chen , Y. Gai , S. Xie , and J. Mi . 2021. “GLC_FCS30: Global Land‐Cover Product With Fine Classification System at 30 m Using Time‐Series Landsat Imagery.” Earth System Science Data 13: 2753–2776. 10.5194/essd-13-2753-2021.

[ece373822-bib-0087] Zhang, Y. , X. Na , and W. Li . 2024. “Impacts of Climate Changes on the Potential Habitat Suitability of *Grus japonensis* on Migration Routes.” Ecological Indicators 166: 112462. 10.1016/j.ecolind.2024.112462.

[ece373822-bib-0088] Zhao, W. F. , L. P. Zong , and M. J. Wang . 2024. “Spatial Distribution of Nature Reserves in China.” Acta Ecologica Sinica 44, no. 7: 2786–2799. 10.20103/j.stxb.202212103552.

