# Supplementary material for: Ensemble Modeling Reveals Severe Contraction of Dhole's ( Cuon alpinus Pallas, 1811) Suitable Habitat and Future Climate Refugia Across China
Source: Ecol Evol. 2026 Jun 19;16(6):e73822. doi: 10.1002/ece3.73822 (PMC13282164; doi:10.1002/ece3.73822)
Supplement: Supplementary file 1 — Figure S1: Analysis of correlation coefficients of the environmental variables. Table S1: Sources of dholes occurrence. Table S2: Model parameters by algorithm. [file ECE3-16-e73822-s001.doc]

**Table S1.** Sources of dholes occurrence.

Our dataset comprises field survey data collected through camera traps and scats in the northeastern and southeastern regions of the Qinghai-Tibet Plateau. This includes the Xinjiang Altun Mountain National Nature Reserve, Xinjiang Lop Nur Wild Camel National Nature Reserve, the Gansu Annanba National Nature Reserve, and the Gansu Yanchiwan National Nature Reserve in 2021–2024 (Huang et al. 2025; Cong et al. 2024), as well as the Xizang Yarlung Zangbo Grand Canyon National Nature Reserve from 2022-2024 and Yunnan Tongbiguan Provincial Nature Reserve from 2019-2024.

To obtain the additional data, we used the search terms “豺”, “红狼” ,”野犬” , “亚洲野狗” in Chinese, accessed through the China National Knowledge Infrastructure (CNKI, <https://www.cnki.net/),> the National Library of China (http://www.nlc.gov.cn/), the Wanfang database (http://www.wanfangdata.com.cn/), as well as news reports or blog articles from platforms such as Baidu(https://www.baidu.com/) and Weibo (https://weibo.com/). In English, we used the terms “Asiatic wild dog”, “dhole”, and “*Cuon alpinus*” to gather occurrence data from CNKI, the Web of Science (https://www. web of science.com/) and Google Scholar (https://research. google.com/).

We implemented rigorous data quality control measures to ensure the reliability of all occurrence records used in this study (Chen et al. 2025). Literature-derived records were included only if they had accurately documented geographical locations and clear temporal data. News reports and blog-sourced records were scrutinized for spatial consistency against established distribution ranges (Liu, Wang, et al. 2024; Liu, Ji, et al. 2024; Huang et al. 2025) and required verifiable visual evidence, such as photographs or videos. Records failing to meet these validation criteria, including those with questionable provenance or insufficient supporting evidence, were excluded from the dataset to maintain analytical integrity.

| **Sources 1: Field surveys** | | |
| --- | --- | --- |
| **ID** | **Time** | **Description** |
| 1 | 2023-2024 | Camera trap and line transect surveys, Xinjiang Altun Mountain National Nature Reserve |
| 2 | 2022-2024 | Camera trap and line transect surveys, Xinjiang Lop Nur Wild Camel National Nature Reserve |
| 3 | 2020-2024 | Camera trap and line transect surveys, Gansu Annanba National Nature Reserve |
| 4 | 2023-2024 | Camera trap, Gansu Yanchiwan National Nature Reserve |
| 5 | 2024 | Camera trap and line transect surveys, Mangya city, Delingha city, Tianjun and Wulan county |
| 6 | 2022-2024 | Camera trap, Xizang Yarlung Zangbo Grand Canyon National Nature Reserve |
| 7 | 2019-2024 | Camera trap, Yunnan Tongbiguan Provincial Nature Reserve |
| **Sources 2: Related scientific research and literature** | | |
| **ID** | **Time** | **Description and References** |
| 1 | 1987 | The Carnivora of Mammalia in China (Gao 1987 ). |
| 2 | 1994 | The Carnivora of Vertebrates in Northwestern China (Zheng 1994 ). |
| 3 | 1997 | Mammalian species in China (China Export-Import Administration Office for Endangered Species 1997). |
| 4 | 1995-2000 | Survey on key terrestrial wildlife resources in China (Ma and Zhang 2009). |
| 5 | 2014 | Wildlife at water sources on the northern slope of the Altun Mountains (Xue et al. 2014). |
| 6 | 2014-2016 | Camera-trapping surveys of the mammal and bird diversity in Wolong National Nature Reserve (Shi et al. 2017). |
| 7 | 2015-2016 | Mammal and Bird Species from Camera Trap Data in Yanchiwan National Nature Reserve (Zhang et al. 2018). |
| 8 | 2018 | Temporal niche relationship between snow leopard (*Panthera uncia*) and its sympatric large carnivores in Qilian Mountains (Zhang et al. 2023). |
| 9 | 2017-2019 | Mammal diversity in the Gansu Anxi Extreme-arid Desert National Nature Reserve (Bao et al. 2020). |
| 10 | 2017-2019 | Mammals Diversity, Distribution and Activity Rhythm in Taiyang River Provincial Nature Reserve (Shao 2020). |
| 11 | 2017-2019 | Diversity and Activity Rhythm on Terrestrial Birds and Mammals in Nangunhe River Basin (Wang 2020). |
| 12 | 2005-2020 | The diversity of large- and medium-sized terrestrial mammals and birds in the Giant Panda National Park (Tian et al. 2021). |
| 13 | 2018-2019 | Species diversity and vertical distribution characteristics of birds and mammals on the western slope of northern Gaoligong Mountain (Wang et al. 2025). |
| 14 | 2018-2020 | Study of Diversity and Spatial Association of Mammals and Land Fowls in Gansu Liancheng National Nature Reserve (Su 2022). |
| 15 | 2020-2021 | Avian and mammal diversities and their altitudinal and seasonal distribution patterns in Yarlung Zangbo Grand Canyon (Shi et al. 2023). |
| 16 | 2019-2022 | Population Survey for *Cuon alpinus* in the Yanchiwan National Nature Reserve of Gansu (Ma et al. 2023). |
| 17 | 2019-2022 | Spatiotemporal distribution patterns of large and medium-sized mammals in Gaoligongshan (Hu et al. 2025). |
| 18 | 2021-2022 | Mammals and birds diversity based on camera trapping in Qimantag Mountain of Altun Mountain (Wang et al. 2024). |
| 19 | 2023 | Dietary composition and niche partitioning of sympatric carnivores in Altun Mountain National Nature Reserve (Cong et al. 2024). |
| 20 | 2020-2023 | Distribution status, occupancy, and spatiotemporal pattern of large carnivores in Dingqing County (Li et al. 2024). |
| 21 | 2005-2024 | Historical and current distribution of dhole (*Cuon alpinus*) in Sichuan Province (Liu, Ji, et al. 2024). |
| 22 | 2016-2024 | The current distribution and prediction of suitable habitat of dhole (*Cuon alpinus*) in Qilian Mountains (Liu, Wang, et al. 2024). |
| 23 | 2021-2024 | Spatial and temporal relationships among Wolf, Dhole, and Red fox (Huang et al. 2025). |
| 24 | 2023-2024 | Diversity of Mammals and Birds by Using Infrared Camera Technology—Taking Menglun Nature Reserve (Song et al. 2025). |
| **Sources 3: News reports or articles in the blog** | | |
| **ID** | **Time** | **Description and References** |
| 1 | 2023 | Hetian, Xinjiang Uygur Autonomous Region. https://baijiahao.baidu.com/s?id=1784965033258584648&wfr=spider&for=pc; https://tieba.baidu.com/p/8400118386?pn=1 |
| 2 | 2019 | Yadong, Tibet Autonomous Region. https://tieba.baidu.com/p/6229571099 |
| 3 | 2013 | Dayi, Sichuan Province. https://cdbpw.chengdu.gov.cn/cdslyj/c110451/2013-03/27/content_ccd53d4923dd4594939c8e05d6c59e6c.shtml |
| 4 | 2023 | Ganzizhou, Sichuan Province. https://baijiahao.baidu.com/s?id=1786426076805928633&wfr=spider&for=pc |
| 5 | 2021 | Xishuangbanna, Yunnan Province. https://baijiahao.baidu.com/s?id=1706142673006082160&wfr=spider&for=pc |
| 6 | 2021 | Gongshan, Yunnan Province. https://m.thepaper.cn/baijiahao_14440696 |
| 7 | 2023 | Tengchong, Yunnan Province. http://yn.people.com.cn/n2/2023/0208/c372456-40293926.html |
| 8 | 2019 | Qilianshan, Qinghai Province. https://baijiahao.baidu.com/s?id=1632927085105708691&wfr=spider&for=pc |
| 9 | 2017 | Sunan, Gansu Province. https://www.gscn.com.cn/gsnews/system/2017/04/06/011668176.shtml |
| 10 | 2017 | Langqian, Qinghai Province. https://society.huanqiu.com/article/9CaKrnK3MtY |
| 11 | 2019 | Dulan, Qinghai Province. http://www.qhio.gov.cn/system/2019/05/13/012876995.shtml |
| 12 | 2021 | Menyuan and Tianjun, Qinghai Province. http://www.qhnews.com/newscenter/system/2021/08/08/013431969.shtml |
| 13 | 2021 | Yushu, Qinghai Province. https://mp.weixin.qq.com/s/CSCNco_wnbObWP40zxjIKw |
| 14 | 2021 | Mangya, Qinghai Province. https://www.chinanews.com.cn/sh/2021/04-18/9457579.shtml |
| 15 | 2022 | Haidong, Qinghai Province. https://www.cnr.cn/qhfw/gstjqh/20220616/t20220616_525866500.shtml |

**References**

Bao, X. K., L. Wang, M. J. Lu, et al. 2020. “Investigation of Bird and Mammal Diversity in the Gansu Anxi Extreme‐Arid Desert National Nature Reserve Using Infrared Camera Traps.” Biodiversity Science 28, no. 9: 1141–1146. (in Chinese). https://doi.org/10.17520/biods.2020089.

Chen, K., W. Shao, Y. Li, et al. 2025. “Biomod2 Modeling for Predicting Suitable Distribution of Bamboo Bat (Tylonycteris pachypus) Under Climate Change.” Animals 15: 1164. https://doi.org/10.3390/ani15081164.

China Export‐Import Administration Office for Endangered Species. 1997. Distribution of Mammalian Species in China. China Forestry Publishing Press (in Chinese).

Cong, W., Y. Zhang, T. F. Huang, et al. 2024. “Dietary Composition and Niche Partitioning of Sympatric Carnivores in Altun Mountain National Nature Reserve.” Acta Theriologica Sinica 44, no. 6: 695–705. (in Chinese). https://doi.org/10.16829/j.slxb.150941.

Gao, Y. T. 1987. Fauna Sinica Mammalia. Vol. 8. Carnivora. Science Press (in Chinese).

Hu, W. Q., X. Y. Li, K. O. Onditi, et al. 2025. “Spatiotemporal Distribution Patterns of Large and Medium‐Sized Mammals in a Biodiversity Hotspot: Implications for Conservation.” Biological Conservation 301: 110863. https://doi.org/10.1016/j.biocon.2024.110863.

Huang, T. F., Y. Zhang, X. M. Liu, et al. 2025. “Spatial and Temporal Relationships Among Wolf (*Canis lupus*), dhole (*Cuon alpinus*), and Red Fox (*Vulpes vulpes*).” Acta Ecologica Sinica 45, no. 13: 6600–6608. (in Chinese). https://doi.org/10.20103/j.stxb.202407151650.

Li, X. Y., Y. J. He, X. E. Chen, et al. 2024. “The Distribution Status, Occupancy, and Spatiotemporal Pattern of Large Carnivores in Dingqing County, Xizang Autonomous Region.” Acta Theriologica Sinica 44, no. 6: 681–694. (in Chinese). https://doi.org/10.16829/j.slxb.150943.

Liu, K., S. N. Ji, T. P. Guan, and S. Li. 2024. “A Preliminary Study of the Historical and Current Distribution of Dhole (*Cuon alpinus*) in Sichuan Province.” Acta Theriologica Sinica 44, no. 6: 804–814. (in Chinese). https://doi.org/10.16829/j.slxb.150981.

Liu, Y. L., Y. D. Wang, Y. B. Li, et al. 2024. “The Current Distribution and Prediction of Suitable Habitat of Dhole (*Cuon alpinus*) in Qilian Mountains, China.” Acta Theriologica Sinica 44, no. 6: 749–761. (in Chinese). https://doi.org/10.16829/j.slxb.150969.

Ma, F., and J. L. Zhang. 2009. Survey on Key Terrestrial Wildlife Resources in China. China Forestry Publishing House.

Ma, Z. B., P. Wang, Y. B. Li, et al. 2023. “Population Survey for *Cuon alpinus* in the Yanchiwan National Nature Reserve of Gansu.” Journal of Gansu Forestry Science and Technolgy 48, no. 3: 57–59. https://doi.org/10.3969/j.issn.1006-0960.2023.03.012.

Shao, Y. P. 2020. Mammals Diversity, Distribution and Activity Rhythm in Taiyang River Provincial Nature Reserve, Yunnan. Master's Degree of Yunnan Normal University. (In Chinese). https://doi.org/10.27459/d.cnki.gynfc.2020.000257.

Shi, X. G., Q. Hu, J. Q. Li, et al. 2017. “Camera‐Trapping Surveys of the Mammal and Bird Diversity in Wolong National Nature Reserve, Sichuan Province.” Biodiversity Science 25, no. 10: 1131–1136. (in Chinese). https://doi.org/10.17520/biods.2017193.

Shi, X. Y., X. Y. Li, C. Y. Wei, et al. 2023. “Avian and Mammal Diversities and Their Altitudinal and Seasonal Distribution Patterns in Yarlung Zangbo Grand Canyon, China.” Biodiversity Science 31: 22491. (in Chinese). https://doi.org/10.17520/biods.2022491.

Song, Z. Y., B. Wang, L. Z. Bai, et al. 2025. “Survey on the Diversity of Mammals and Birds by Using Infrared. Camera Technology‐Taking Menglun Nature Reserve in Xishuangbanna, Yunnan Province as an Example.” Journal of Anhui Agricultural Sciences 53, no. 7: 80–85. (in Chinese). https://doi.org/10.3969/j.issn.0517-6611.2025.07.014.

Su, T. W. 2022. Study of Diversity and Spatial Association of Mammals and Land Fowls in Gansu Liancheng National Nature Reserve, China. Doctor Degree of Beijing Forestry University. (In Chinese). https://doi.org/10.26949/d.cnki.gblyu.2022.000012.

Tian, J., S. Y. Zhu, X. F. Zhang, et al. 2021. “The Diversity of Large‐ and Medium‐Sized Terrestrial Mammals and Birds in the Giant Panda National Park: A meta‐Analysis Based on Camera‐Trapping Data.” Biodiversity Science 29: 1490–1504. (in Chinese). https://doi.org/10.17520/biods.2021165.

Wang, J. H., Y. D. Lv, Y. F. Zhao, Y. Xiong, Y. H. Fang, and R. X. Wang. 2025. “Species Diversity and Vertical Distribution Characteristics of Birds and Mammals on the Western Slope of Northern Gaoligong Mountains. Journal of Sichuan Forestry.” Science and Technology 46, no. 1: 48–56. (in Chinese). https://doi.org/10.12172/202409250002.

Wang, X. L., J. Q. Xu, S. F. Zhang, H. Li, and J. Li. 2024. “A Preliminary Survey of Mammals and Birds Diversity Based on Camera Trapping in Qimantag Mountain of Altun Mountain National Nature Reserve, Xinjiang.” Arid Land Geography 47, no. 10: 1662–1673. (in Chinese). https://doi.org/10.12118/j.issn.1000-6060.2024.426.

Wang, Z. H. 2020. Analysis of Diversity and Activity Rhythm on Terrestrial Birds and Mammals in Nangunhe River Basin, China. Master's Degree of Yunnan University. (In Chinese). https://doi.org/10.27456/d.cnki.gyndu.2020.001590.

Xue, Y. D., F. Liu, T. Z. Guo, L. Yuan, and D. Q. Li. 2014. “Using Camera Traps to Survey Wildlife at Water Sources on the Northern Slope of the Altun Mountains, China.” Acta Theriologica Sinica 34, no. 2: 164–171. (in Chinese). https://doi.org/10.16829/j.slxb.2014.02.008.

Zhang, C. C., J. Wang, J. S. Alexander, et al. 2018. “Biodiversity Assessment of Mammal and Bird Species From Camera Trap Data in Yanchiwan National Nature Reserve, Gansu Province, China.” Journal of Resources and Ecology 9, no. 5: 566–574. (in Chinese). https://doi.org/10.5814/j.issn.1674-764x.2018.05.014.

Zhang, C. Z., T. Ma, L. J. Wu, and X. M. Liu. 2023. “Temporal Niche Relationship Between Snow Leopard (*Panthera uncia*) and Its Sympatric Large Carnivores in Qilian Mountains, Gansu Province.” Acta Theriologica Sinica 43, no. 1: 109–115. (in Chinese). https://doi.org/10.16829/j.slxb.150632.

Zheng, S. W. 1994. Fauna of Rare and Endangered Species of Vertebrates in Northwestern China. China Forestry Publishing House (in Chinese).
